# Safeguarding the Therapeutic Alliance: Managing Disaffiliation in the Course of Work With Psychotherapeutic Projects

**DOI:** 10.3389/fpsyg.2020.596972

**Published:** 2021-02-10

**Authors:** Aurora Guxholli, Liisa Voutilainen, Anssi Peräkylä

**Affiliations:** Faculty of Social Sciences, University of Helsinki, Helsinki, Finland

**Keywords:** therapeutic alliance, disaffiliation, disagreement, psychotherapeutic project, collaborative move

## Abstract

Therapeutic alliance is a central concept in psychotherapeutic work. The relationship between the therapist and the patient plays an important role in the therapeutic process and outcome. In this article, we investigate how therapists work with disaffiliation resulting from enduring disagreement while maintaining an orientation to the psychotherapeutic project at hand. Data come from a total of 18 sessions of two dyads undergoing psychoanalytic psychotherapy and is analyzed with conversation analysis. We found that collaborative moves deployed amidst enduring disagreement can assist the therapist in furthering the disagreement as part of the ongoing psychotherapeutic project. Relying on their collaborative format, therapists utilize collaborative moves to temporarily mend the disaffiliation without necessarily changing their position and re-affiliating with the patient. We show how the relation between the therapist and the patient gets transformed in the moment-by-moment work accomplished in the psychotherapeutic talk.

## Introduction

In conversation analytic studies, the term affiliation is used to describe actions with which a recipient displays that s/he supports the affective stance of the speaker (Lindström and Sorjonen, 2013) or, as Jefferson (2002) put it, that s/he is on the same side with the speaker. An affiliative action is exemplified in the following spate of talk, where recipient (J) affiliates with speaker (M) by strongly agreeing with the latter’s assessment.


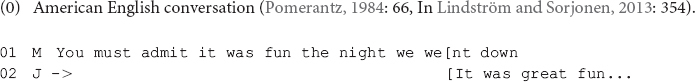


Because of their supportive nature, affiliative actions have a pro-social character (Stivers et al., 2011) and foster social solidarity (Lindström and Sorjonen, 2013).

In a well-known study on storytelling, Stivers(2008: 37) showed how recipients affiliate with the storyteller through responses that support and endorse the teller’s stance, where stance means “the teller’s affective treatment of the events s/he is describing.” In a similar vein, Heritage (2011) investigated recipients’ responses to their co-participant’s telling of a personal experience and showed how emotional first-hand experiences invite others to produce an evaluation by affirming its meaning and nature, thus affiliating with the teller’s stance toward the experience. The affiliative strength of the response, he argued, is determined by the capacity of the response type to convey that the recipient is tuning in to the experience and one way to do it is by actively participating in its articulation. These findings shed light on how being “with” someone requires not only sharing the same epistemic stance on their personal experiences but also endorsing the displayed affect and emotion (Peräkylä and Sorjonen, 2012; Ruusuvuori, 2013).

In their review on affiliation in conversation, Lindström and Sorjonen (2013) argued that context can play a crucial role in shaping and constraining affiliative displays, distinguishing between ordinary and institutional encounters where affiliation can have diverse relevancies. For example, Ruusuvuori (2005) investigated trouble-telling sequences in healthcare consultations and found a very different pattern compared to ordinary conversation. In her study, the majority of professionals displayed no affiliation to troubles-telling patients and, when they did, they prioritized the patient by focusing on his/her experience, without disclosing their own.

In psychotherapy, affiliation has been investigated as a responsive action by the therapist endorsing the preferences realized in the client’s prior utterance (Muntigl et al., 2013). Focusing on relational stresses in Emotion-focused Therapy (Greenberg et al., 1993; Greenberg, 2002), Muntigl and Horvath (2014) found that in order to re-affiliate, the therapist retreats from his/her position and joins with the client’s position brought up in his/her disaffiliative response. Re-affiliation, they observed, can be achieved both verbally (by utilization of discursive markers of agreement or formulations) and non-verbally (nodding). Prosody is another important means to achieve affiliation. In a study on the prosodic aspects of therapists’ empathic communication, Weiste and Peräkylä (2014) showed how therapists’ formulations of clients’ descriptions of emotions can lead up to two different trajectories of interaction: one validating the client’s emotional description and the other evaluating and challenging it. The difference, they found, lies in the prosodic features of the formulation, with the validating trajectory characterized by prosodic continuity whereas the challenging trajectory characterized by prosodic disjuncture.

**FIGURE 1:**
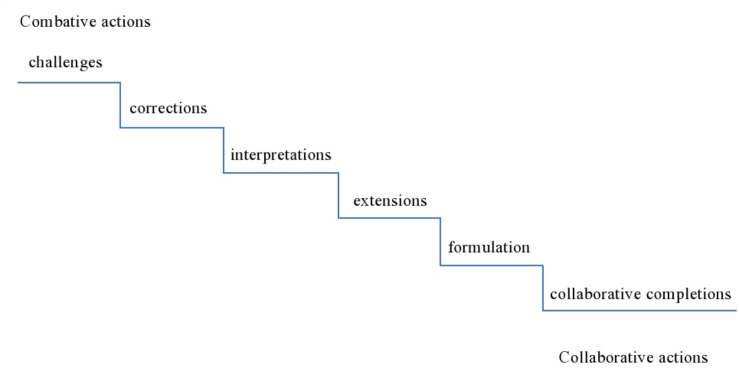
Gradient of therapist’s combative to collaborative practices (Antaki, 2008).

Antaki(2008: 27) defined formulations as the most (ostensibly) cooperative practice used by therapists to “display their grasp of, and present an alternative to, the client’s accounts of their experiences.” Visualizing the therapist’s practices in a descending gradient from more combative to more cooperative moves (Figure 1), he placed formulations at the *more cooperative* end of the slope, where *cooperative* refers to practices that are designed in such a way that shows that the therapist is cooperatively following the line of the client’s account (Ibid., 30). Other cooperative/collaborative^1^ practices investigated in conversation analytic studies include extensions and collaborative completions. Similar to formulations, extensions are a powerful means to display intersubjectivity (Vehviläinen, 2003). Therapists can use extensions as a way to show to the patient that they hear and understand what s/he is saying (Sacks, 1992: 58). In conversation analytic studies of mundane interaction, collaborative completions are a third practice where speakers construct the turn collaboratively, with the subsequent speaker pre-emptying completion of the previous speaker’s turn constructional unit (Sacks et al., 1974). Other less affiliative actions are interpretations, corrections and challenges (Antaki, 2008).

Therapists use both supporting and challenging actions to assist the patient in moving forward from the current capacity to accommodate innovative moments or new experiences to a potential greater capacity (Ribeiro et al., 2013). While supporting actions confirm and validate the client’s experience, challenging actions move beyond client’s maladaptive self-narratives. In their work, therapists make moment-by-moment decisions on how to guide the clients to perceive alternative perspectives (Greenberg and Safran, 1987; Lomas, 1987; Ribeiro et al., 2013). This moment-by-moment work transpires in therapeutic projects, defined as “interactional projects^2^ with accompanying therapeutic aims” (Peräkylä, 2019: 273).

For the successful implementation of a therapeutic project, it is important that the patient goes along the therapist’s suggested interactional direction (Schegloff, 2007). Patients can put the therapeutic alliance at risk in a number of ways. For example, they can misalign with the therapist’s interactional project by steering the interaction in diverging directions (Voutilainen et al., 2010) or disaffiliate with the therapist by not endorsing his/her understanding of the client’s situation (Muntigl and Horvath, 2014; Muntigl, 2020). Likewise, therapists might also undermine the therapeutic alliance and disaffiliate with the client by not responding empathically to the client’s prior talk (Muntigl and Horvath, 2014) or by challenging her/him with strong oppositional statements displaying unsupportive disagreement. Weiste (2015) defined the latter as therapists maintaining their divergent perspectives, disregarding the clients’ claim as unrealistic and claiming privileged access to the clients’ domain of knowledge. In response to unsupportive disagreement, clients react with irritation and anger. Such ruptures put the accomplishment of the therapeutic project at risk by straining the therapeutic relation.

Ruptures can however be worked through in a number of ways. One way for therapists to mend a rupture is by displaying supportive disagreement. Weiste (2015) showed how, in psychoanalysis and cognitive psychotherapies, therapist’s supportive disagreement implies work at finding congruence with the client’s perspective, validating the client’s emotional experience, and respecting his/her epistemic primacy. Such supportive disagreement, in turn, prompts clients to confirm and elaborate their experience. In a similar context of disagreement, Muntigl et al. (2013) found that in Emotion-focused therapy, talk is organized in such a way that therapists maintain affiliation by neutralizing potential conflict and preserve client’s epistemic primacy or experience by privileging their viewpoint. As these findings show, the relevancies and displays of affiliation vary not only among the different contexts in which the interaction occurs, but also within various approaches to one type of institutional context, being psychotherapy.

While it is widely accepted that the role of the therapeutic bond is central to the psychotherapeutic process and positive outcomes (Horvath and Bedi, 2002; Orlinski et al., 2003), how this bond is formed and maintained at the interactional level remains understudied (Lepper and Mergenthaler, 2007). In psychoanalysis, Loewald(1960: 16) proposed that it is the significant interactions between patient and analyst which ultimately lead to structural changes in the patient’s personality. The aim of this study is to enhance our understanding of how participants orient to the therapeutic relation during the moment by moment unfolding of the therapeutic work. To this end, we focus on one particular psychotherapeutic approach, being psychoanalytic psychotherapy, and investigate how therapists work with disaffiliation resulting from enduring disagreement. The focus of our work is twofold: (a) to describe how therapists deploy collaborative moves amidst enduring disagreement as part of their work with the therapeutic relation; and (b) to show how these collaborative moves while aiming to soothe disaffiliation, are not necessarily affiliative in nature and do not indicate re-affiliation on behalf of the therapist. In this way, we show how therapists seek to maintain the therapeutic alliance at a safe place by not necessarily being always on the same side with the patient.

## Data and Method

Data come from a total of 18 sessions of two dyads undergoing psychoanalytic psychotherapy. The first dyad (10 sessions) is at the end of their second year of the psychoanalytic process. The therapist is a woman in her early forties and the patient a man in his late twenties. The second dyad (8 sessions) is at the very beginning of the psychoanalytic process. The therapist is a woman in her late twenties and the patient is a woman in her mid-thirties. Each session lasts 50 min, amounting to a total of 15 hours of interaction. We video recorded the sessions during 2016-2017 and obtained informed consent from both therapists and patients. No statement of the ethics of the research design was requested from the University of Helsinki Ethical Review Board in the Humanities and Social and Behavioral Sciences as the study does not meet the requirements specified by the Finnish National Board on Research Integrity on ethics approval. All names and other identification potential details in the data extracts are altered.

It is worth mentioning here that in Albania, it is common practice that, in psychoanalysis, the patient sits (instead of lying down) in a 45-degree angle with the therapist. Another difference with the traditional psychoanalytic practice regards the frequency of the meetings, with the first dyad meeting every other week, whereas the second every week. To distinguish between conventional psychoanalysis and its adjusted format, we refer to the practice in our data as *psychoanalytic psychotherapy*.

Data was analyzed with conversation analysis (CA). As a first step in the analytic procedure, the recordings were listened to a number of times. We first collected all the instances in which the therapist deploys a collaborative move – a total of 117 (56 from the first dyad and 61 from the second one). We identified *collaborative moves* based on Antaki’s (2008) gradient of therapist’s more combative to more collaborative practices, with the more collaborative including practices used to display that the therapist is collaboratively following the line of the patient’s account. These practices included:

(1) *Collaborative completions* (16) defined as the pre-emptive completion of one speaker’s turn constructional unit by a subsequent speaker (Sacks et al., 1974); they can be produced as an affiliating utterance, built as a continuation of the turn-in-progress and as a completion of that turn (Lerner, 1991).
(2) *Formulations* (11) defined by Heritage and Watson (1979, 1980) as actions that propose a version of events following the previous speaker’s own account but introduce a transformation.
(3) *Extensions* (5) referring to a speaker extending the previous speaker’s turn as a way to promote a further account of what the patient is saying (Vehviläinen, 2003).

As the therapist’s actions are more combative or more collaborative in their format and not necessarily in their local force (Antaki, 2008: 27), next we focused on the interactional environment in which these moves are deployed. In a second revision, we regrouped the therapist’s collaborative moves based on the type of environment in which they were deployed, focusing mainly on the ones deployed amidst environments of disagreement – a total of 32 (22 from the first dyad and 10 from the second one). We considered disagreement to be a significant environment for the therapeutic relation as it fosters disaffiliation and can impede the implementation of the therapist’s interactional project at hand.

Next, we demarcated the stretch of talk within which the collaborative move occurs, starting from the moment when the disagreement between the therapist and the patient first emerges, following its escalation until the deployment of the collaborative move by the therapist, up to the therapist’s restating her position on the issue at stake. These stretches of talk were then transcribed using CA transcription conventions (Jefferson, 2004). Our analysis focused on the sequential function that collaborative moves play in managing disaffiliation.

## Therapist’s Utilization of Collaborative Moves

We found that one way for therapists to foster the ongoing affiliation with the patient is to make use of a collaborative move. On the other hand, when deployed amidst disagreement, a collaborative move can be used to soothe the disaffiliation. In this section we show four instances where therapists deploy a collaborative move to either foster the ongoing affiliation or soothe the disaffiliation resulting from enduring disagreement.

### Collaborative Move Deployed Amidst Affiliation

One type of environment in which the therapist deploys a collaborative move is when she and the patient are affiliated, meaning they share the same affective stance with regards to what they have been talking about so far. Such a move can foster the ongoing affiliation which in turn, with the therapeutic relation being at a safe place, allows the therapist to advance the interactional project. The following talk is an example of one such use of a collaborative move (indicated in all extracts with an arrow). It is extracted from a mid-session section of dyad I. The patient is talking about his recent plans to start a new music band. Toward the end of a story telling sequence on his previous bands, the patient mentions the name of his second band, “blind spot.” Extract I shows what happens next.


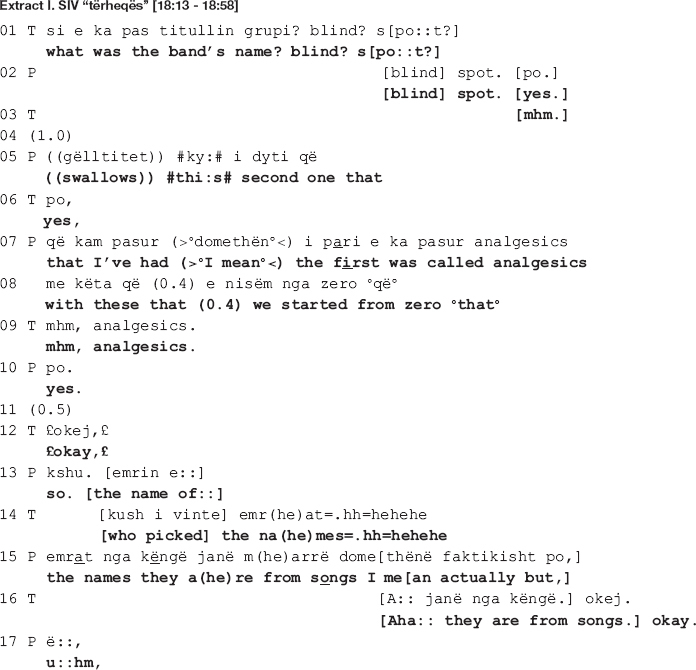



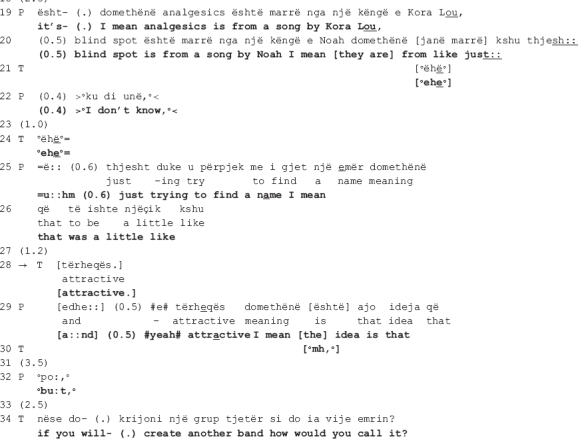


In the beginning of the extract, the therapist topicalizes the band name, expanding the sequence by means of a repair initiation (line 1). The patient accepts the shift in focus of talk and grants the information requested (line 2) but soon after goes back to the story of his first band “with these that we started from zero” (lines 7-8), orienting to his previous elaboration as in need of an uptake by the therapist. The latter, however, pursues her interactional project, being exploration of the new topic (band names) by first recycling the name of the band (line 9), followed by another inquiry into the authors of the names (line 14). The therapist’s talk is produced in smiley voice, culminating in laughter, conveying a sympathetic stance toward the band names. In response, the patient aligns by granting the information required (lines 15, 19-20) and also affiliates with the therapist by partaking in the amusement through shared smiles and laughter (lines 12, 14, and 15).

In line 21, by means of a continuer (Schegloff, 1982) produced immediately following a transition relevant place (Schegloff, 2007), the therapist invites the patient to continue talking. The patient, however, displays difficulties in completing his turn (notice the extended sound in “just:” at the end of line 20; the short 0.4 s pause and the filler “I don’t know” produced in increased speed and low volume in line 22; the gap in line 23; all these accompanied by a hand gesture indicating word searching). In response, the therapist produces another continuer (line 24), this time orienting to the patient’s turn as incomplete by declining a relevant uptake following the gap in line 23. The patient picks up his account by recycling the last word “just” in the beginning of the turn, making a second attempt at completing it (lines 25-26: = *u:hm (0.6) just trying to find a name I mean that was a little like*). Similar difficulties are displayed here as well, when in the beginning of the turn he produces a prolonged filler “u:hm,” followed by a 0.6 s long pause; another filler “I mean” at the end of the utterance; and a 1.2 s long gap (line 27). Also, a similar hand gesture indicating word searching accompanies the difficulties in producing the talk.

In response to the patient’s displayed difficulties, the therapist “helps out” the patient by completing his turn (line 28: *[attractive.]*). She provides a candidate word which the patient displays difficulties in producing. While the patient orients to his previous turn as complete (see how line 29 starts with “and”: *[a:nd] (0.5) #yeah# attractive I mean [the] idea is that*), the therapist, on the other hand, orients to it as incomplete. By means of a “helpful utterance completion” (Ferrara, 1994) she supplies a candidate word which qualifies the band names and completes the patient’s turn. The patient confirms by producing first a minimal agreement token “yeah,” next a repetition of the word “attractive” (line 29). In addition to offering lexical help, the therapist explicates content at risk of being left unsaid by the patient (Koivisto and Voutilainen, 2016). This content is of relevance to her interactional project of exploring the band names, which she has explicitly pursued thus far in the talk and will continue to do so (line 34). Lastly, the content at stake matches with her previously displayed sympathetic stance toward the names of the bands, now explicitly referring to them as “attractive.”

### Collaborative Move Deployed Amidst Disaffiliation

Advancing an interactional project might not always be an easy task for the therapist. Disaffiliation resulting from enduring disagreement is one type of environment in which the therapist and the patient share different affective stances with regards to a topic of talk. As the following analysis of extracts II and III will reveal, deployment of a collaborative move amidst such an environment aims at soothing the disaffiliation which in turn, with the therapeutic relation being temporarily restored, allows the therapist to advance the interactional project at hand.

#### Collaborative Move Deployed Amidst Covert Disagreement

The following extract is from the same dyad (I). The therapist and the patient are talking about the patient’s need for therapy. In the beginning of the session, the patient tells at length about his recent engagements with a series of new activities, depicting himself as open minded, willing to take up new challenges, open to new experiences, in short, a person of many talents. In response, the therapist questions his need for therapy. The patient does not answer the question, in its place attributing the recent positive changes in his behavior to therapy. The talk in extract II (a) below shows what happens next, when the therapist pursues her interactional project, inviting the patient once more to elaborate on his need for therapy. Here we see how, despite the patient’s alignment with the therapist’s project, the therapeutic relation is nevertheless put at risk as an ostensibly long-standing disagreement resurfaces, conducing to disaffiliation. In what follows, we first show how the disagreement transpires (extract II a), next how the therapist attempts at soothing the disaffiliation by deploying a collaborative move (extract II b).


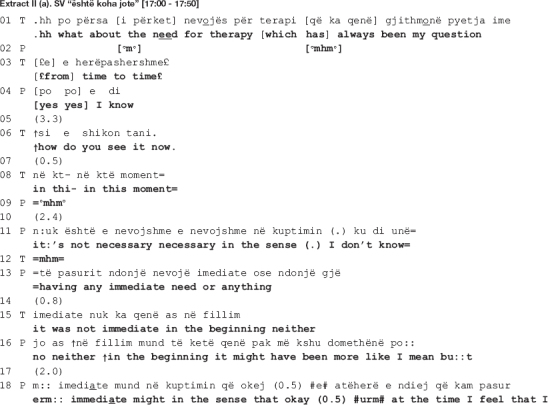



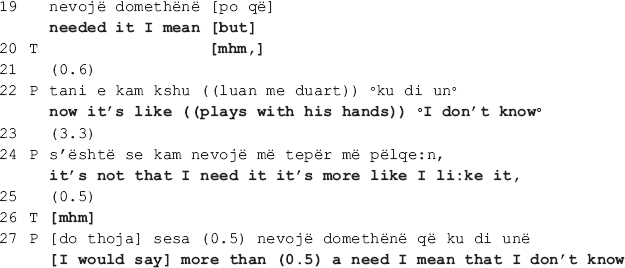


The talk above reveals that the patient’s need for therapy is a recurrent topic among this dyad: the therapist accounts for her turn as having “always been [her] question” (line 1), and the patient in line 2 first acknowledges it (Goodwin, 1980; Jefferson, 1983), then explicitly confirms it (line 4). Moreover, this topic seems to be an issue of long-standing dispute as the therapist and the patient orient to each other’s stances as conveying opposite viewpoints. In the beginning of the extract, the therapist questions the patient’s need for therapy and invites him to elaborate on the topic (lines 1, 2, 6, and 8) [this is the therapist’s second attempt, the first one - not shown here - occurring right before the above stretch of talk]. The patient indicates that he understands the question (see the acknowledgment tokens in lines 2 and 4, and also the confirmation in line 4) yet delays the response for quite some time (see the gaps in lines 5, 7, and 10). The dispreferred response (Schegloff, 2007) is then designed in such a way that by mitigating his need for therapy (“not necessary;” “not immediate”), the patient avoids both claiming that he needs therapy which would be in open disagreement with the therapist but also that he doesn’t need it which would be incongruent with his own stance (lines 11 and 13).

Despite the patient’s interactional work to avoid overt disagreement, the therapist does not endorse his stance. What is more, she openly confronts him by rejecting his claim as incorrect (line 15). The patient responds immediately with a *pro forma* answer which soon transforms into a mitigated response (lines 16, 18-19), displaying a clear orientation toward avoidance of overt disagreement. In response to the therapist’s continuous lack of endorsement of the patient’s stance (see the gap in line 23), the latter proceeds with a new claim, being that it is not out of need but rather “it’s more like [he] likes it” (line 24) that he comes to therapy, conveying thus lack of willingness to bring the therapeutic process to an end, a natural implication of admitting that he has no need for therapy.

In the next approximately 1.5 min, the patient accounts for what he finds beneficial and enjoyable in therapy, concluding that although it is not necessary, he would nevertheless like to continue with it [data not shown here]. This final remark produced right before extract II (b) reveals that the disagreement concerns the broader implication of the need for therapy, being the patient’s continuation of therapy, which he seems to be in favor of. While the patient indicates that he has not changed his stance, there is no indication of the therapist having changed hers either, the disagreement remaining thus pending in the air as they enter the ensuing talk. The therapist’s collaborative move under scrutiny here transpires amidst such moment of enduring disaffiliation. Its local function, as the analysis will reveal, is to soothe the disaffiliation so that the therapist can proceed with the interactional project at hand.


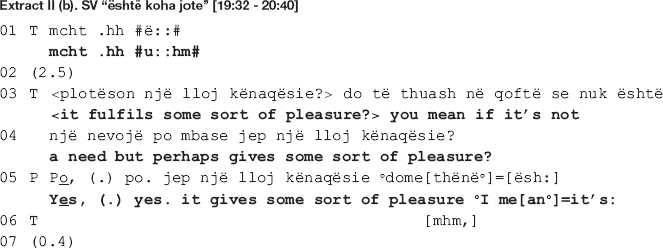



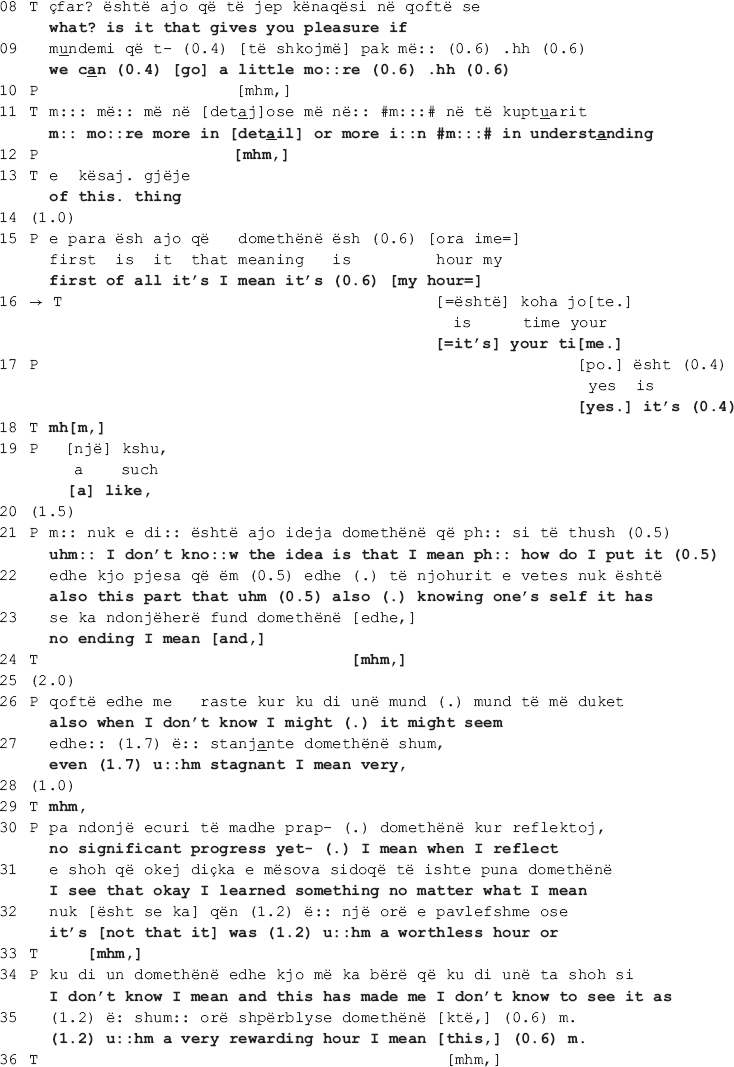


In the beginning of the extract (lines 3-4), the therapist expands the prior sequence [not shown here] by opening up an other-initiated repair sequence addressing trouble with understanding the patient’s response (Schegloff, 2007). By topicalizing “pleasure,” she accepts the patient’s shift in focus of talk from the need for therapy to him liking and enjoying it. Such a move is already a first step toward collaboration; the therapist displays an orientation toward accepting the patient’s reason to continue with therapy and alters her interactional project accordingly. She leaves the exploration of the patient’s need for therapy behind and moves on to invite exploration of therapy as a pleasure fulfilling experience. This attempt is nevertheless not very successful as instead of aligning with the therapist’s altered project, the patient orients to it as somewhat problematic.

The patient first confirms straightaway by producing the affirming particle “yes” twice, followed by a repetition of the last part of the therapist’s previous turn “gives some sort of pleasure” (line 5). His immediate confirmation however seems to orient more toward the collaborative nature of the therapist’s move than the assertion itself. In his response, the patient treats this assertion as problematic in some way. Incompatible with the immediate and rather strong confirmation, the patient displays thinking, treating the therapist’s assertion as news hence not what he meant (he gazes away from the therapist while prolonging the vowels of the verb *is* here translated as “it’s:”). Moreover, by delaying the elaboration made relevant in the therapist’s post-expansion, the patient orients to it as a dispreferred. As Schegloff (2007) argues, a preferred response would have been a sequence-closure relevant in which the therapist endorses the patient’s stance in regard to his need for therapy, therefore reaffiliating with him.

In pursuit of her interactional project, the therapist makes a second attempt at getting the patient to expand his answer, this time using a wh-question (lines 8-13: *what? is it that gives you pleasure if we can (0.4) [go] a little mo::re (0.6).hh (0.6) m:: mo::re more in [detail] or more i::n #m:::# in understanding of this. thing*), working as a specific expansion elicitor (Muntigl and Zabala, 2008). In response, the patient produces a first part of his answer though not without difficulties (*first of all it’s I mean it’s (0.6) [my hour* = *]*). Prior to responding, there is a 1-s-long gap (line 14), whereas while responding, the patient gazes away from the therapist (line 15, up to producing the words “my hour”); he pauses for 0.6 s before the production of the first list item while making back and forth short head movements indicating searching; he accompanies the word search by making round circles with his hands, as an illustration of the mental process he is going through. The therapist acknowledges the success of this second move by responding collaboratively *([* = *it’s] your ti[me.*).

In line 16, where the target action of our analysis is deployed, the therapist utilizes a highlighting formulation (Weiste and Peräkylä, 2013), showing that she is collaboratively following the line of the patient’s account. Remember that the therapist and the patient are disaffiliated when entering the talk. By means of this collaborative move, the therapist works to soothe the disaffiliation in two ways. Firstly, by latching the formulation onto the patient’s prior turn, the therapist produces it very similarly to a helpful utterance completion (Ferrara, 1994), supplying the vocabulary item the patient displays difficulties in finding. In this way, she not only acknowledges the difficulties but also accepts the answer (notice how the turn is prosodically produced with a closing intonation). Secondly, by formulating “it’s my hour” (line 15) as “it’s your time” (line 16), the therapist displays understanding of the patient’s answer and, at the same time, receipt of it. While producing the turn, she gazes away from the patient, toward her left-hand side, and accompanies the gaze with a wide hand gesture. Both gestures indicate a cognitive process in progress, most probably including recalling as the patient has already mentioned the word “hour” in his previous talk taking place right before extract II (b) [data not shown here] saying that what he likes about therapy, among other things, is that it is his hour. In this way, she shows that she has been attentive to his talk and remembers what he has said.

The patient rushes to confirm (line 17: *[yes.] it’s (0.4)*) yet, instead of item listing initiated prior in the talk (line 15), he proceeds with a transformative answer (Stivers and Hayashi, 2010), retrospectively transforming the focus of the question’s agenda from the pleasure he derives from therapy to going back to talking about its benefits (lines 21-35). While the therapist attempts at repairing disaffiliation through stretching out a hand at collaboration for the ensuing talk, the patient treats the disaffiliation as in need of resolution rather than merely soothing, pursuing the therapist’s endorsement of his stance. Misaligning with the therapist’s interactional project, he bypasses the topic of therapy as a pleasure fulfilling experience and goes back to accounting for how he benefits from therapy, hence his need for it (lines 21-35). In what follows, the focus remains on the patient’s needs that are fulfilled in therapy, as described by the patient. The therapist aligns with the patient’s diverging interactional project and does not go back to questioning neither his need for therapy nor the pleasure it fulfils. In this way, she contains further escalation of disagreement by leaving the differences in their positions behind, extending her collaboration to include the patient’s control over the ensuing talk.

#### Collaborative Move Deployed Amidst Overt Disagreement

Sometimes, the disagreement between the therapist and the patient is more overt, including a persistent pursuit of the interactional project by the therapist. The talk in overt disagreement is more explicit and both patient and therapist openly affirm their different positions on the matter. In the following stretch of talk a collaborative move is deployed amidst one such environment. It is extracted from the very beginning of a session from the second dyad. The session starts with the patient asking the therapist how she has been doing, adding that while the focus is always on her, human kindness necessitates reciprocation. The therapist first finds it difficult to answer, next produces a short response, and soon after diverts the focus of talk on the patient, stating that this is the time and place to talk about her. The patient agrees and following a gap of 4 s, the talk ensues as shown below.


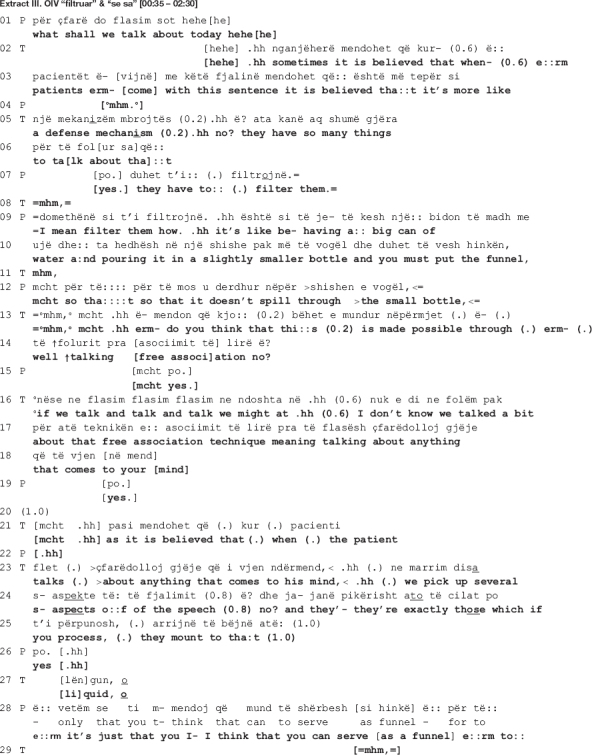



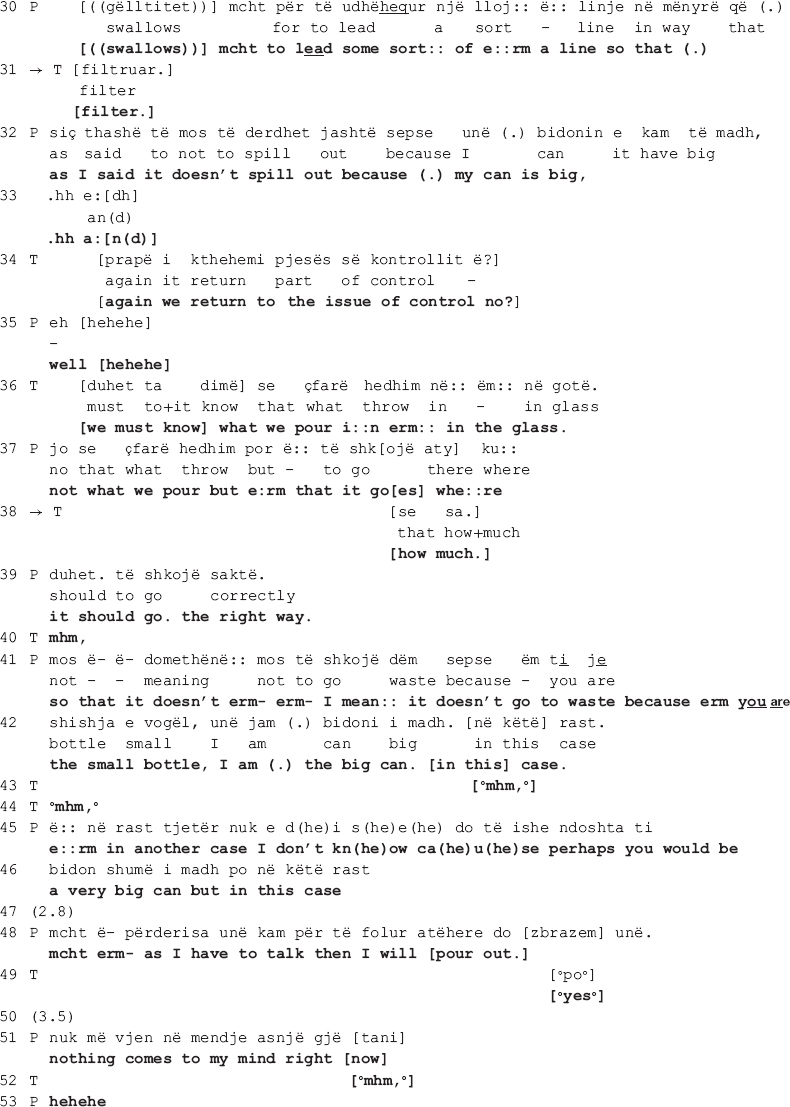


In the beginning of the extract, the patient asks for help from the therapist to pick a topic of talk (line 1). The therapist interprets the patient’s request as a defense mechanism (lines 2-3 and 5-6) implying that she has a lot to say but can’t due to psychological barriers. By declining to help, the therapist is not only engendering a dispreferred action (Schegloff, 2007), she is also disagreeing with the patient’s implied claim that she doesn’t know what to talk about. To appease the combative potency of the interpretation, the therapist (a) mitigates the temporal validity of her interpretation (see the use of “sometimes” in the beginning of the turn, line 2); and (b) attributes the interpretation to external referents: *the therapists* (notice how the turn is formatted in passive voice: “it is believed that,” line 3) and *the patients* (as opposed to this one therapist interpreting this one patient’s behavior).

The patient interrupts and following a *pro forma* response (see the agreement token “yes” in line 7 in response to the therapist’s use of the question tag “no,” which can also be translated as “isn’t it” in English, in line 5) proceeds to complete the therapist’s turn. Claiming her right to know about patients’ experiences by merit of being a patient herself (Pomerantz, 1980), she starts talking about what patients need, “filtering” in this case (line 7). Despite seemingly in agreement with the therapist, the patient disagrees by attributing her difficulties in picking a topic not to her internal psychological barriers but rather to the lack of a funnel that will help her in filtering her talk (lines 7, 8-9, and 11).

In her response in line 13 onward, the therapist invites the patient to freely associate when talking in therapy by elaborating on how free association is conducted and what its therapeutic aim is. Here again the patient responds *pro forma*, seemingly in agreement with the therapist (lines 15, 19, 26) to only go back to the funnel metaphor, this time explicitly asking the therapist to “serve as a funnel” (lines 28-30: *e:rm it’s just that you I- I think that you can serve [as a funnel] e:rm to: [((swallows))] mcht to lead some sort: of e:rm a line so that (.)).* It is in this moment of their talk, amidst enduring disagreement, that the therapist produces two collaborative moves, orienting to the disaffiliation as in need of soothing. In the first move (line 31: *[filter.]*), the therapist recycles the patient’s word “filter” (first mentioned in line 7). Overlapping with the patient’s swallowing, the therapist hurries up to give her the word she thinks the patient is looking for (see how she uses a filler “e:rm” and prolongs both the filler and “to:” in line 28). By recycling the patient’s own word, the therapist shows that she has not only heard but also understood what the patient previously said. As the patient declines to elaborate, an action made relevant by the therapist’s invitation to freely associate when talking in therapy, this first collaborative move treats the ongoing disaffiliation as in need of soothing.

The patient however declines the therapist’s “help” and sequentially deletes the collaborative completion of her turn (line 30: *[((swallows))] mcht to lead some sort: of e:rm a line so that (.)*). In accounting for why she needs the therapist to serve as a funnel, the patient is not only declining the latter’s invitation to associate freely but also restating her different position on the matter. In response, the therapist makes another interpretation, this time attributing the patient’s position to her controlling tendencies (lines 34 and 36: *[again we return to the issue of control no?] [we must know] what we pour i:n erm: in the glass.*). The patient corrects her interpretation (line 37: *not what we pour but e:rm that it go[es] whe:re*) to which the therapist responds with a second collaborative move (line 38: *[how much.]*), a collaborative completion (Lerner, 1991) of the patient’s turn following word searching (notice the use of filler “e:rm” in line 37). In what follows, the patient continues to account for her position, while the therapist makes no further attempts at pursuing her interactional project in which the patient would associate freely. The therapist does not go back to the issue under dispute, thus neither reaffirming her position nor confronting the patient’s.

Similar to what happens in extract II, the therapist’s collaborative moves do not imply re-affiliation with the patient. She and the patient remain disaffiliated throughout the talk, and the collaborative moves deployed here demonstrate the therapist’s orientation toward soothing the disaffiliation. The difference, however, lies in the fact that here the therapist pursues her interactional project more persistently by proceeding from implicit to more explicit talk, openly affirming her position on the matter. Her collaborative moves orient to the disaffiliation as in need of soothing yet reaffiliation is not achieved as neither she nor the patient endorses the other’s position.

#### Collaborative Move Deployed to Further the Disagreement

So far, we have shown how therapists make use of a collaborative move as a means to either foster the ongoing affiliation or soothe the disaffiliation, in both cases maintaining an orientation toward furthering the interactional project at hand. The analysis of its local function reveals that when deployed amidst disaffiliation resulting from enduring disagreement, the collaborative move does not necessarily indicate that the therapist is re-affiliating with the patient. In extracts II and III, we saw how, soon after the collaborative move, the therapist goes back to her disagreeing stance. Hence the practice of “helping out” the patient is a demonstration that the therapist has been with him/her all along paying attention to what s/he has been saying and thus understanding his/her talk rather than an endorsement of his/her opposing stance. In two instances in our collection, this is even more so the case as the therapist does both actions within the same turn: (1) attempts at collaboratively completing the patient’s turn while at the same time (2) goes back to her stance. Here we show one of these instances. As the stretch of talk leading to this move is fairly long, we first show how the move is designed and its sequential position (Extract IV (a)). Next, we show the longer version of the stretch of talk where the move is deployed, which allows us to analyze its local function, being the therapist’s orientation toward advancing her interactional project (Extract IV (b)).

### Design and Sequential Position of the Collaborative Move

The talk here is extracted from a mid-session section from the first dyad. The therapist and the patient are talking about the patient’s recent dreams.


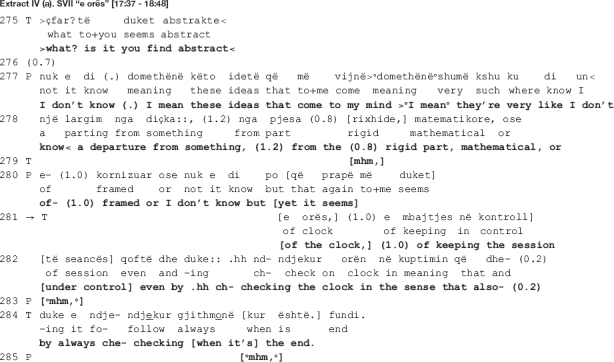


In the beginning of the extract, the therapist takes issue with the patient’s rejection of her previous interpretation as “too abstract” [data not shown here], openly confronting him (line 275). In response, the patient initiates another attempt at elaborating on the therapist’s interpretation (lines 277-280) to only abandon it halfway, going back to rejecting it (line 280). It is right before the projected upcoming of the rejection that the therapist comes in with the move under scrutiny here.

The therapist’s turn in lines 281-282 and 284 is designed as collaborative completion of the patient’s previous turn. The therapist hooks her turn into the patient’s previous one: she recycles the preposition “e” at turn initial position and produces the rest of the turn as a grammatical continuation of the patient’s. This proposition may be used as an adjective initial particle (the patient ends up using it as such, the adjective being “e kornizuar” in Albanian, translated in English as “framed”) but also as an initial particle indicative of the genitive case (the therapist makes such use of it, the genitive case of the word “orë” (“clock” in English) being “e orës” (of the clock)). In Albanian language, the noun comes before the adjective, thus the therapist turn becomes: “… a departure from something, from the (part) rigid, mathematical, or of the clock, of keeping the session under control …”

As the patient’s turn is produced with notable difficulties, the therapist attempts at soothing the ongoing disaffiliation by helping out the patient and giving him the word he seems to be searching for. Nevertheless, as the subsequent analysis will reveal, what is structurally constructed as a collaborative move – an extended hand at a moment of need – turns out to be a reaffirmation of the therapist’s previous disagreeing position. In the next section, we show how the disagreement emerges and escalates.

### Local Function of the Collaborative Move

In this session, the therapist and the patient talk about the patient’s two recent dreams. During the first 8 min, the patient describes his dreams; for the rest of the session, the therapist and the patient engage in interpreting the dreams together. A close look at the interpretation sequences in this session reveals a recurring pattern of more or less the following organization: (a) first either the therapist or the patient topicalizes an element from the dreams; next the therapist invites the patient to elaborate on its meaning using the free associations technique; the patient either does not elaborate, engaging instead in dream telling, or initiates elaboration but does not establish connections, meaning or explanations, either case giving way to misalignment; (b) in her next move, the therapist suggests an interpretation with which the patient openly disagrees or agrees minimally, either way not elaborating on it as made relevant by the therapist’s action initiation, resulting in disaffiliation.

A similar pattern can be observed in the extract below where the therapist and the patient are talking about a fourth element from the dream, “the academic writing guy.” In the dream, the patient’s academic writing lecturer appears as his therapist and continuously interrupts him. His statistics lecturer also appears at some point in the dream, asking him to go and see his therapist, inferring the academic writing lecturer. The therapist invites the patient to freely associate on who the “academic writing guy” might resemble to. Again, the patient fails to elaborate and, as the therapist pursues an answer, the patient finally claims to not have one, saying that he can’t find any resemblance to “some concrete person.” A long silence of 5 s ensues before the talk proceeds as shown in extract IV (b) below.

In the beginning of the extract, the therapist suggests that one possible interpretation might be that the academic writing guy resembles the patient himself (line 256). As in the previous extract, the interpretation is mitigated (the interrogative format frames the turn as hypothetical; uncertainty markers are incorporated in the talk, i.e., the epistemic modal auxiliary “can” and the 0.2 s pause; the turn is uttered in a soft tone of voice), orienting to the epistemic asymmetry with regards to access to the patient’s inner experience (Weiste et al., 2016). The patient agrees partially and hesitantly, naming parts of himself that might resemble to what the notions of “academic writing” and “statistics” represent: rigidness (line 259) and stubbornness (line 266). In this moment in the talk, the therapist and the patient are both affiliated and aligned, as they share the same stance (the therapist produces several agreement tokens in lines 260, 263, 267, and 273, accompanied by nodding throughout the lines 260-262) and the patient is engaged in the same interactional project with the therapist, beginning to elaborate on the therapist’s suggested meaning within the same real-world referential frame.


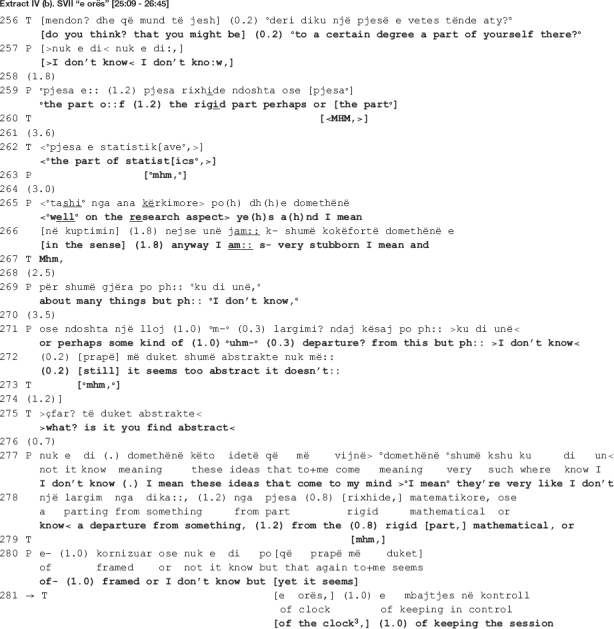



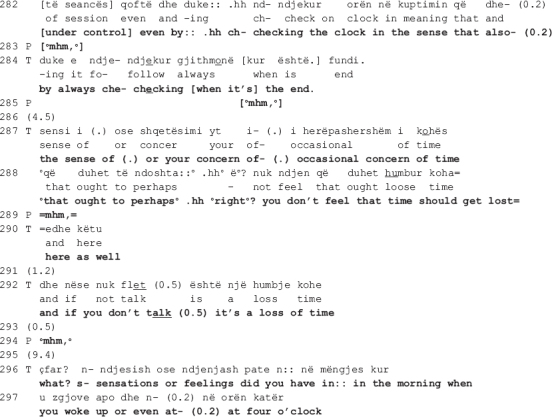


Their affiliation is, however, short-lived. The patient does not succeed in establishing a meaningful link between the element in the dream and his real-life world personality trait, “rigidness.” He ends up rejecting the therapist’s interpretation as “too abstract,” one which “doesn’t …” (line 272) possibly *convince* or *make sense* to him. The therapist questions the acceptability of the patient’s answer (line 275: >*what? is it you find abstract*<) as she responds with an understanding check that functions as a repair initiation (Schegloff et al., 1977) pursuing expansion (Muntigl and Zabala, 2008). Similar to findings from other CA studies of psychotherapy when patients do not respond to therapist’s action in a manner that is relevant to the interactional goals (Muntigl and Zabala, 2008; Koivisto and Voutilainen, 2016), the therapist orients to the patient’s response as resisting her interpretation without elaborating on his grounds for not endorsing it and, what is more, declining to produce an alternative interpretation. The therapist’s confrontational turn is produced with irritation (the talk is speeded up, her problem with understanding is not accounted for, and her gaze is stern), and so is the patient’s (he looks away from the therapist, shrugs his shoulders in a quick and tense gesture, and frowns).

The patient starts to expand his answer by unpacking “these [abstract] ideas that come to [his] mind” (line 277). In an attempt at elaborating on the meaning of the dream, he produces a three-part list (Jefferson, 1990) of candidate descriptors of his personality traits which he might be departing from (lines 278, 280: *a departure from something, (1.2) from the (0.8) rigid [part,] mathematical, or of- (1.0) framed or I don’t know but [yet it seems]*). The first item, “rigid,” is recycled from his own previous talk (line 259); the second one, “mathematical,” bears similarity to the therapist’s “the statistics’ part” (line 262); a third new item, “framed” (line 280), is added before he ends the listing only to go back to his previous position as projected by the use of the contrastive “but,” possibly heading toward *abstract*. Overall, the patient’s turn is produced with hesitation: it is embedded with uncertainty markers (notice the recurrent use of the knowledge disclaimer “I don’t know,” the filler “I mean,” and the self-repair initiations) as well as visible difficulties in producing the words (vowels are prolonged at the end of a TCU and the in-turn silences are also long). The entire turn is accompanied by hand gestures indicating word searching, shrugging and gazing away from the therapist, all pointing to the patient being engaged with necessary cognitive work to produce the answer.

It is in this moment of the talk when the therapist’s interactional project is critically stalled that she comes back to their previous disagreement with a seemingly collaborative move, to only reassert her stance with regards to the patient’s “sense of control” as a plausible interpretation of him having lost the watch in the dream (data not shown here). In line 281, the therapist intervenes right before the patient reiterates his disagreement, interrupting him as soon as he utters the contrastive “but.” Though produced not immediately after the patient’s word searching (see the long pause of 1 s in line 280, accompanied by hand gesture indicating searching), the therapist’s turn is designed as to collaboratively complete the patient’s previous turn (lines 281-282, 284: [*of the clock*^3^] *(1.0) of keeping the session [under control] even by.hh ch- checking the clock in the sense that also- (0.2) by always che- checking [when it’s] the end.*). Similar to what happens in extracts II and III when the patient’s turn is produced with notable difficulties, here as well the therapist seems to attempt at soothing the disaffiliation by giving him the word he is searching for. Nevertheless, what is structurally constructed as a collaborative move turns out to be a return to her disagreeing position.

By seemingly adding to his list of candidate descriptors of his personality traits, what the therapist actually does is bring forth evidence of how his “sense of control” is exhibited in the therapy. The evidence has a three-fold function: (a) the therapist comes back to her previous stance, affirming once more that there is a meaningful connection between the watch and the time, and losing the watch might mean that the patient let go of his controlling tendency (of the time in this case); (b) she strengthens her interpretation by offering evidential grounds for it: “keeping the session under control even by checking the clock … by always checking when it’s the end” (lines 281-282; 284), and “you don’t feel that time should get lost here as well” (lines 288, 290); and (3) accounts for the unacceptability of the patient’s previous answer (the understanding check in line 275) by explicating “the abstract” relation between a personality trait (rigid, mathematical, framed) and how it is demonstrated in therapy (what the patient actually does as a result of possessing the trait), a relation the patient did not elaborate upon.

The therapist grounds her position on the therapeutic setting, a physical reality to which both have access to and where the patient’s overt behavior is exhibited. In further escalating the disagreement, the therapist maintains an orientation toward the interactional project of dream interpretation. The patient however withdraws from engaging in further talk and the therapist accepts his disengagement by moving on to a new topic (lines 296-297). They agree to leave their opposing views behind and move on to another interactional project. In this way, further escalation of disagreement is contained, allowing for the therapeutic work to resume.

## Discussion

This study sheds light on how the psychotherapeutic process takes place through sequentially organized patterns of talk. We have focused on one particular realm of experience-under-transformation in psychotherapy, the *relation* between therapist and patient (Peräkylä, 2019). This study revealed one way in which therapists in psychoanalytic psychotherapy attempt at mending relational ruptures while maintaining an orientation to the therapeutic work. We showed how locally collaborative actions can assist therapists in pursuing the disagreement as part of the ongoing psychotherapeutic project, while momentarily mending the arising disaffiliation with the patient. Relying on the sequential properties of collaborative moves, therapists can show their patients that they have been carefully listening to them and understand their perspective. However, these helpful behaviors do not necessarily imply re-affiliation with the patient. What they do is earn the therapist the right to hold on to her/his position and even come back to it if the issue at stake is of therapeutic relevance. By clearing out the way of potential mishearing and/or misunderstanding of the patient’s view, the therapist legitimizes her/his right to sustain the disagreement while, at the same time, acknowledges the necessity and importance of remedying the relational rupture. In this way, the therapist maintains simultaneous orientation toward the therapeutic work and the relation with the patient, constantly balancing between therapeutic projects and relational dynamics.

These findings correspond with Sacks’ argument that, in conversation, attempts at “coming to an understanding” is one way to deal with disagreement (1973). Schegloff (lecture XV) quotes Sacks having said that ‘conflict does not arise because people do not understand each other. It’s that the first way, the first line of defense for dealing with conflict is to turn it into a problem of understanding or even hearing.’ While in our study the therapist displays understanding as a means to hold on to her/his position, in other studies we see a similar orientation toward making sure a shared understanding has occurred before disagreeing with the patient. For example, Koivisto and Voutilainen (2016) showed how one practice that therapists use to not endorse the client’s answer is to deploy a disaffiliative candidate understanding (Antaki, 2012). Where acknowledgment or validation is made relevant, therapists initiate repair as a way to legitimately pass the opportunity to affiliate with the client without openly challenging the later.

However, coming to a shared understanding is not always possible. As extracts in this and other studies disclose (see for example Voutilainen et al., 2018) often times in psychotherapeutic talk the therapist and patient do not sort out the disagreement by coming to an agreement but rather by accepting that they have diverging viewpoints and moving on to a new therapeutic project. Such orientation to disagreement suggests that, when the balance between the therapeutic work and the therapeutic relation is at risk, therapists tend to privilege the later. This inclination toward safeguarding the relation resonates with findings from studies of human interaction which reveal an overall tendency toward solidarity and cooperation (e.g., Clayman, 2002; Tomasello, 2008). In a study of laughter in complaint sequences, Holt (2012) found that recipients of complaints use laughter to display a somewhat disaffiliative stance with the teller and misalign with the activity by contributing to topic termination while subtly maintaining social concordance.

Just as disagreement and conflict might put the solidarity at risk, mere displays of being “with” the patient can withhold the therapeutic work. Although a general level of affiliation needs to be maintained throughout the therapeutic work in order to secure the patient’s commitment to therapy, it is important for the patient to learn to move safely and freely between moments of affiliation and disaffiliation rather than being persistently stuck in one or the other position (Peräkylä, 2019: 273). This study explicated how “momentary transformation of relation” (Peräkylä, 2019: 271) as one realm of experience targeted in psychotherapy takes place amidst such moments and how therapeutic aims intertwine with interactional projects in the moment-by-moment work accomplished in the psychotherapeutic talk. While the present study investigated one particular practice (collaborative move) deployed amidst one specific type of interactional environment (disagreement), much remains to be investigated regarding various degrees of collaboration displayed by each such move or other types of environments that put the therapeutic relation at risk. Likewise, unearthing other practices that therapists deploy to address ruptures in therapeutic alliance can further inform our understanding of how patients’ transformation of experience takes place in psychotherapy.

## Data Availability Statement

All datasets presented in this study are included in the article.

## Ethics Statement

Ethical review and approval was not required for the study on human participants in accordance with the local legislation and institutional requirements. All participants provided their written informed consent to participate in this study.

## Author Contributions

AG collected, transcribed, and translated the data, and wrote the article. All authors contributed in data analysis.

## Conflict of Interest

The authors declare that the research was conducted in the absence of any commercial or financial relationships that could be construed as a potential conflict of interest.

## Footnotes

1 In conversation analytic literature, the terms *cooperative* and *collaborative* are used interchangeably. To avoid confusion, from here on in the text we will use the term *collaborative*.

2 Schegloff(2007: 244) defined interactional project as “a course of conduct being developed over a span of time (not necessarily in consecutive sequences) to which co-participants may become sensitive, which may begin to inform their inspection of any next sequence start to see whether or how it relates to the suspected project, theme, stance, etc.”

3 The word “orë” in the Albanian language means both “watch” and “clock.” In extract 4 (a), the patient lost his wristwatch whereas in here, the therapist is referring to the wall clock in the therapy room.

## Appendix A. CA Transcription Conventions

D: Speaker identification: for example Doctor (Dr), Patient (P), Mother (M)
[ ]  Brackets: Onset and offset of overlapping talk
= Equal sign: No gap between two utterances
(0.0) Timed pause: Silence measured in seconds and tenths of seconds
(.) A pause of less than 0.2 seconds
. Period: falling or terminal intonation
, Comma: level intonation
? Question mark: rising intonation
↓ Rise in pitch
↑ Rise in pitch Fall in pitch
- A dash at the end of a word: an abrupt cutoff
< Immediately following talk is ‘jump started’, starts with a rush
><  Faster-paced talk than the surrounding talk
<>  Slower-paced talk than the surrounding talk
____  Underlining: some form of stress, audible in pitch or amplitude
HI  Capital letters: talk that is louder than the surrounding talk
: Colon(s): Prolongation of the immediately preceding sound
°°   Asterisks surrounding a passage of talk: talk with lower volume than the surrounding talk
.hh  A row of ‘h’s prefixed by a dot: an inbreath
hh  A row of ‘h’s without a dot: an outbreath
# Number signs surrounding a passage of talk: spoken in a ‘creaky’ voice (vocal fry)
£ Smiley voice
@ Animated voice
